# First-principles investigation of polytypic defects in InP

**DOI:** 10.1038/s41598-022-24239-w

**Published:** 2022-11-16

**Authors:** Christian Dam Vedel, Søren Smidstrup, Vihar P. Georgiev

**Affiliations:** 1 Device Modelling Group, James Watt School of Engineering, University of Glasgow, Glasgow, UK; 2 Synopsys Denmark ApS, Copenhagen, Denmark

**Keywords:** Engineering, Materials science, Nanoscience and technology, Physics

## Abstract

In this paper we study polytypic defects in Indium Phosphide (InP) using the complementary first-principles methods of density functional theory and non-equilibrium Greens functions. Specifically we study interfaces between the ground state Zincblende crystal structure and the meta-stable Wurtzite phase, with an emphasis on the rotational twin plane defect, which forms due to the polytypic nature of InP. We found that the transition of the band structure across the interface is anisotropic and lasts 7 nm (3.5 nm). Due to this, a crystal-phase quantum well would require a minimal width of 10 nm, which eliminates rotational twin planes as possible quantum wells. We also found that for conducting current, the interfaces increase conductivity along the defect-plane ([11$\bar{2}$]), whereas due to real growth limitations, despite the interfaces reducing conductivity across the defect-plane ([111]), we found that a high degree of polytypic defects are still desirable. This was argued to be the case, due to a higher fraction of Wurtzite segments in a highly phase-intermixed system.

## Introduction

The transistors, the workhorse of micro-electronics, have seen numerous design changes and optimisations throughout the last 50 years^1^. Most of these optimisations have been aimed at reducing the channel length or increasing the flow of charge carriers in the channel by other means, such as straining the silicon^2^. These optimisations have resulted in faster, smaller and more energy efficient transistors and hence electrical circuits and electronic devices.

In recent years the number of transistors on a chip has reached a level, where power dissipation has become a major issue which hamper further miniaturisation^3^. To alleviate this problem, a lower operating voltage is required, but this in turn raises issues with the switching speed. One possible solution to this heat dissipation problem, is the usage of high-mobility channel materials, such as the compounds formed from the group III and V elements (aka. III–Vs)^4,5^.

The field of Integrated Photonic Circuits (IPCs) has followed a miniaturisation trend similar to the transistors in electronic circuits, but progress has halted due to low optical confinement in waveguides^6^. A possible solution to this confinement issue, that is currently being researched, is the integration of InP membranes around the active and passive components. These membranes act similar to silicon dioxide in Silicon On Insulator (SOI) devices, i.e. they isolate the device from the substrate and thereby improve the performance of the device^7^.

In fact various III–V materials are widely used in a number of devices for photonic applications, ranging from lasers to fiber optics, to detectors^8,9^, and even more exotic ones like topological microlasers^10^ and meta-stable lasing microdiscs^11^.

Integration of III–Vs into the current silicon platform usually yields a large number of crystal defects, due to the lattice mismatch between the silicon substrate and the III–V materials. A significant amount of research has been done, to investigate how to avoid the formation of defects, such as threading dislocations^12^ or rotational twin planes^13,14^. A similarly large amount of research has been made on the effect of such defects on the semiconductor and device performance^15–18^. Usually, defects in any materials constituting a device, leads to a degradation of the device’s performance^19^. However, if the defects can be controlled, they can potentially be beneficial for the device’s performance, by enhancing key figures of merit such as the drive current. As an example, rotational twin planes (aka. stacking faults) in Gallium Nitride (GaN), are suspected to work as atomically thin, shallow, quantum wells, which could be used as optical sensors at specific wavelengths^20–24^. Hence controlled formation of defects, such as these twin planes in GaN, can potentially lead to novel new devices.

In order to explore the possibility of using defects in III–V materials to improve device characteristics, we specifically investigate rotational twin planes in bulk InP in this paper. Our aim is firstly to investigate the effect of these defects on the current flow in the semiconductor. Secondly, we will explore the possibility of these defects also functioning as quantum wells in InP. The research was carried out by numerical simulations, using the complimentary benefits of the first-principles (ab initio) method Density Functional Theory (DFT), combined with Non-Equlibrium Greens Functions (NEGF), as implemented in the state-of-the-art simulation software suite QuantumATK from Synopsys^25–27^. Details regarding the simulation methodology can be found in the “Methods” section.

**Figure 1:**
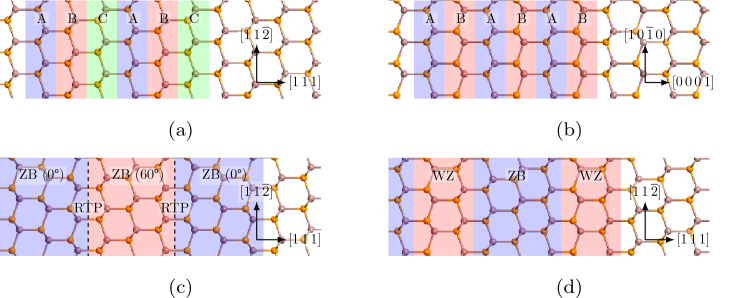
The 4 different kinds of InP systems investigated in this paper. (**a**) Pristine Zincblende (ZB). (**b**) Pristine Wurtzite (WZ). (**c**) A Rotational Twin Plane (RTP), here shown with a periodicity of 1 RTP every 3 layers of ZB. (**d**) A mixture of WZ and ZB, here shown with a periodicity of 2 layers WZ every 3 layers ZB.

InP is a direct band gap III–V material, that crystallises in the diamond-like Zincblende (ZB) phase, which is shown in Fig. 1a. It has also become possible in recent years, to grow InP in the meta-stable hexagonal Wurtzite (WZ) phase, which is shown in Fig. 1b^28^. Due to the low energy separation of these two crystal-phases (approximately 6 meV per atom, according to our simulations), random transitions between ZB and WZ often occurs throughout the growth phase. These transitions are regarded as defects, since they are deviations from the pristine ZB or WZ lattice. The smallest transition defect region in a ZB phase, a single “WZ” layer, is called a Rotational Twin Plane (RTP). A RTP is named as such, because it rotates the ZB region after it by $60^{\circ }$ around the [111]-axis, as compared to the ZB region before it, see Fig. 1c. If two of these RTPs form sequentially, the layers can no longer be viewed as part of separate ZB regions, and a well-defined WZ phase is formed, as shown in Fig. 1d. RTPs are often also called stacking faults, because they are an interruption of the usual ABC-stacking sequence of ZB along the $\langle 111\rangle$-directions. The stacking sequence of ZB and WZ is shown in Fig. 1, together with an RTP super-lattice and a maximally intermixed ZB/WZ system.

## Results

### Pristine bulk Zincblende and Wurtzite InP

The difference between the properties of the ZB and WZ phases of different materials, varies substantially and can be quite significant for some materials. For example, the III–V materials: Aluminium phosphide (AlP), Gallium Phosphide (GaP) and Aluminium Antimonide (AlSb), all have a indirect band gap in their ground state ZB phase, but a direct band gap in their meta-stable WZ phase^29,30^. The band gap size can also change quite substantially, 1eV for example, in the group IV material compound Silicon Carbide (SiC). The reason for these changes can largely be found in their minimal unit cells. The 4-atom unit cell of WZ can be viewed as a folding of the 2-atom unit cell of the ZB phase along the $\langle 111\rangle$ directions. Therefore the conduction band minima at the *L*-point in ZB, is folded to the $\Gamma$-point in WZ, leading to a new “dark” conduction band with $\Gamma _8^c$ symmetry. Optical transitions involving this band is impaired by symmetry, and thus its photoluminescence is lower than the regular “bright” conduction band ($\Gamma _7^c$ symmetry). In some materials, this dark conduction band is energetically lower than the bright conduction band, leading to a so-called pseudodirect band gap. Even more interestingly, in some materials, such as Gallium Arsenide (GaAs), the bands are so close energetically, that transitions between them can be induced by stress^31^.

**Figure 2:**
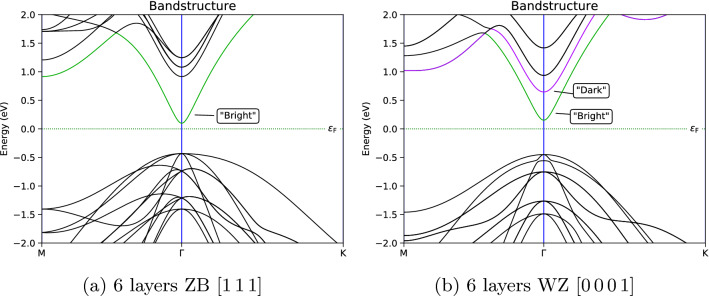
Band structures of InP phases in a hexagonal unit cell.

In order to properly compare the band structures of the ZB and WZ phases of InP, we set up a 12-atom hexagonal supercell of ZB along the [111]-direction. This 6-layer supercell can be readily compared with a triple repetition of the simple 2-layer unit cell of WZ. We then calculated the band structures of these supercells, and the results are shown in Fig. 2. These band structures were calculated without spin-orbit coupling and with a Generalised Gradient Approximation (GGA) exchange-correlation functional. GGA functionals are known to underestimate band gaps, but the other band differences, as well as the overall band structure and trends, are still correct. This was validated by comparison with band structures calculated, using the highly accurate hybrid exchange-correlation functional HSE06, as implemented in QuantumATK. The band structures calculated with HSE06, can be found in the supplementary materials (see Supplementary Fig. 9). The band structure parameters have been extracted, and is displayed in Table 1 for convenience.

**Table 1:** Band structure parameters extracted from the band structures shown in Fig. 2 and Supplementary Fig. 9.

Band parameter	ZB/WZ difference GGA	ZB/WZ difference HSE	ZB GGA	WZ GGA	ZB HSE	WZ HSE
Conduction band edge (eV)	0.05	0.05	0.10	0.15	0.58	0.63
Valence band edge (eV)	0.02	0.02	− 0.43	− 0.45	− 0.73	− 0.75
Band gap (eV)	0.07	0.07	0.53	0.60	1.31	1.38
‘Dark’ conduction band offset (eV)	NA	NA	NA	0.49	NA	0.44

As it can be seen from Fig. 2, the band gaps in both phases of InP were found to be direct, with the band gap of the WZ phase being 70 meV larger than the ZB phase. The same difference was observed with HSE06, and here the ZB band gap value was 1.31 eV, quite close to the experimental band gap of 1.34 eV^32^. The energy difference between the dark and bright conduction band was found to be 0.49 eV (0.44 eV with HSE06). A transition from direct to pseudodirect band gap in InP, would therefore require a very high amount of stress. One interesting feature seen in the band structures in Fig. 2, is the anisotropy of the valence band close to the $\Gamma$-point in ZB. The effective mass along the $\Gamma -K$ path is different from the $\Gamma -M$ path. This anisotropy is not present in the WZ band structure, and is thought to be caused by the symmetry differences between the two phases. In ZB there are uninterrupted bonds along any $\Gamma -M$ path, but this is not the case in the hexagonal WZ lattice.

**Figure 3:**
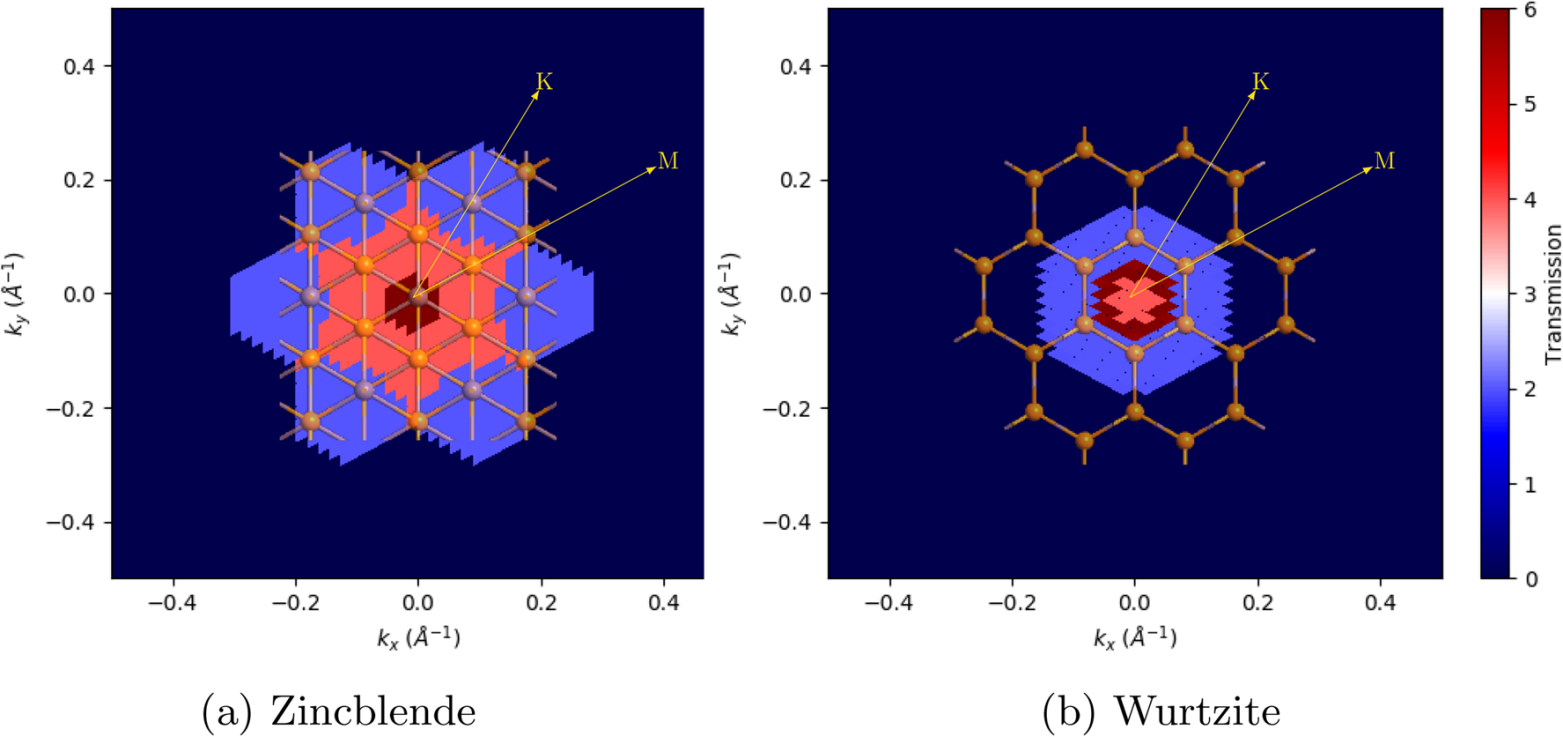
InP transmission coefficients at − 0.66 eV in reciprocal space, with the real space lattice overlaid for comparison of directions.

This valence band anisotropy can also be seen in the hole transport of the transmission spectra, calculated using NEGF, as shown in Fig. 3. The NEGF calculations were set up with the transport directions along [111]/[0001] and without a source/drain bias voltage. It is seen that the transport is less asymmetric in WZ than in ZB, and that both phases favours transport along the M-direction, the direction of the bonds, over the K-direction.

### Rotational Twin Planes as quantum wells

**Figure 4:**
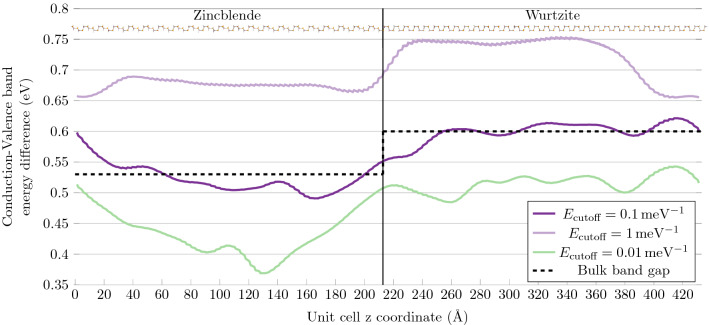
Conduction-valence band energy difference in 127 atomic layer long InP ZB/WZ interface system for three different DOS cutoff values.

It is a common concern, that the defects caused by polytypism, may function as small 70 meV deep quantum wells, despite their sizes of potentially a single layer (in the case of a RTP). The transition between ZB and WZ in these defects, is commonly modelled with an abrupt band gap change at the interfaces between the two phases. Realistically there will be charge transfer between the two phases, and a smooth transition will occur. To investigate how abrupt this transition is, and thereby the validity of treating RTPs as quantum wells, we set up a ZB/WZ interface supercell system, with periodic boundary conditions. We then calculated the Projected Density Of States (PDOS), projected onto the atoms. In the PDOS we used a cutoff of ${0.1} \hbox {meV}^{-1}$, to define the Conduction Band Minimum (CBM) and Valence Band Maximum (VBM). This cutoff was chosen, to replicate the bulk band gap values, in the centres of the phases. We used a system of 127 layers, 64 layers of WZ and 63 layers of ZB, to ensure that the phases would be bulk-like far away from the interfaces. The CBM-VBM difference is plotted in Fig. 4, as a function of the atoms’ z-coordinate (the axis of the stacking sequence i.e., [111]/[0001]). It is plotted for a few different cutoff values, together with the unit cell of the system itself.

From the results presented in Fig. 4, it is evident that the change of the band structure from ZB to WZ, is far from abrupt. We find that the transition between the band structures requires approximately 7 nm or 20 atomic layers. Thus there will be no notable band offset or misalignment in the 1-layer thin RTPs or in segments of a few layers of another phase. This result was tested using systems of shorter length, as well as with the HSE06 functional (see Supplementary Figs. 10 and 11). These calculations confirmed the conclusion obtained here, but with slightly smaller transition lengths (approximately 6 nm and 5 nm). This is likely caused by the enhanced interaction with the periodic image in the shorter system. The reduced tunnelling, due to the higher band gaps, in the system calculated with HSE06, could also be the cause.

Another interesting result, is that the band structure transition is anisotropic. The band energy difference moving from ZB to WZ, is different in comparison to the transition from WZ to ZB. This is probably because of the polarity of InP, due to the in-layer dipole between Indium and Phosphorus atoms. The two sides of a InP layer is electronically different. The WZ to ZB transition only takes approximately 3.5 nm or 10 layers. A well-defined quantum well structure, made from different phases of InP, will therefore require a minimum width of 10.5 nm, to reach the well-phase CBM/VBM difference in the centre of the well. These results were also observed in the work of Dacal and Cantarero^33^, with shorter and smoother transitions. We attribute the differences to their usage of meta-GGA and plane waves to describe local properties.

**Figure 5:**
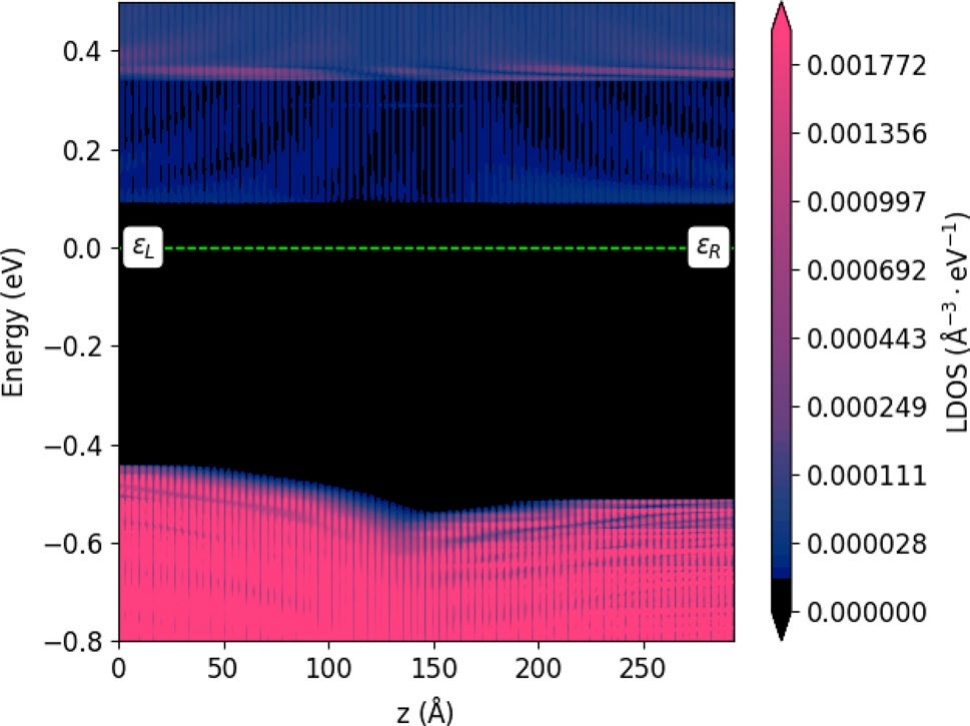
LDOS of InP interface between Zincblende (on the left) and Wurtzite (on the right).

One issue with this investigation, is the influence of the interface in the periodic image of the supercell. This is exemplified by comparing Fig. 4, with the shorter system in Supplementary Fig. 11. To eliminate this possible error, we used the advantage of the semi-infinite electrodes in a NEGF calculation (see the “Methods” section). We therefore converted the ZB/WZ interface system to a NEGF system. We then calculated the Local Density Of States (LDOS), which is shown in Fig. 5.

In the LDOS in Fig. 5, we see the NEGF advantage of the semi-infinite electrodes. Since both electrodes are true bulk systems, they exhibit the ZB/WZ bulk band gaps. They then enforce this value, to the CBM/VBM differences, on the left/right side of the central region. Therefore, if the central region is wide enough, that the bands can properly relax to the electrode values, this calculation is better suited for the transition at a single interface. From this calculation we see that the ZB to WZ CBM/VBM transition, is a lot smoother than indicated by the earlier periodic calculations. We also notice that the transition period is notably longer, approximately 15 nm (from flat band to flat band) as compared to the 7 nm of the periodic calculation. There is also a slight increase in the CBM/VBM difference at the interface, which was not seen in the periodic calculations.

Another point to note, is that the transition is entirely determined by the valence band. The conduction band is flat across the interface. This contrasts the models commonly used to describe interfaces, wherein the CBM and VBM is taken from bulk calculations. In the case of InP, the bulk calculations predict band-misalignment at both the CBM and VBM (see Table 1). Using such models, would therefore predict a drastically different transition, than the one seen here.

It is evident from the VBM-bending in Fig. 5, that hole transport in InP, will be drastically influenced by the formation of ZB/WZ interfaces, such as those in RTPs. Despite the flatness of the CBM across the interface, there are still some reallocation of higher-energy states due to the interface. This could, potentially, give rise to a perturbation of the electron transport in InP.

### Transport through polytopic defects in InP

To investigate the impact of RTPs and other polytopic defects on the hole and electron transport in InP, we set up six different NEGF systems: three systems with a periodicity of 1 RTP every 3, 6 and 9 layers of ZB respectively, one with a 2-layer WZ segment every 3 ZB layers, another with a 4-layer WZ segment every 3 ZB layers and lastly one with a 2-layer WZ segment every 6 ZB layers. In all of these systems, the central regions were also used as the electrodes, so that the systems were fully periodic. Pristine ZB and WZ systems were also set up for comparison. Examples of the systems can be seen in Fig. 1. A small, intrinsic, n-type background doping of $1\times 10^{17}\;\hbox {cm}^{-3}$, was added to the systems, to aid convergence. For more details on the calculations, we refer to the “Methods” section. We then calculated the differential conductivity along the [111]/[0001]-direction. And we plotted it, versus doping level and applied bias voltage, relative to the conductivity of the pristine ZB system:

**Figure 6:**
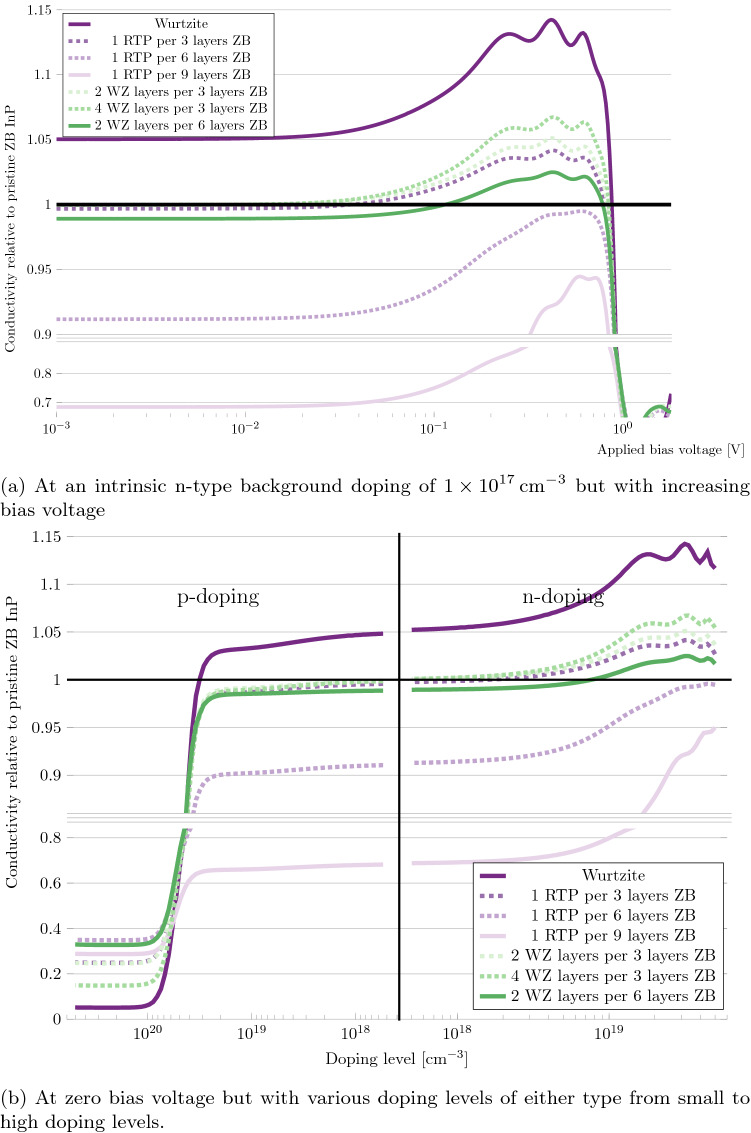
Differential conductivity of InP phase-defect systems along the [111]/[0001] crystallographic direction, relative to a pristine ZB system.

From the results shown in Fig. 6a, we see that in the Ohmic region (the low-bias region where the curve is linear), pristine WZ has a 5% higher conductivity than pristine ZB. We also see that the defects generally decrease the conductivity, in this region. The largest reduction is seen, in the system with the fewest defects, the system with a single RTP every 9 layers of ZB, with a 32% reduction in conductivity. There is likely a trade-off taking place, between the defects perturbing the system, and thereby decreasing the conductivity, and the systems becoming more similar to the pristine WZ system, and thereby increasing the conductivity. By this logic, it makes sense that, the system with a conductivity closest to the pristine WZ system, is the system with the highest fraction of WZ, the system with 4 WZ layers every 3 ZB layers.

The Ohmic region breaks down at approximately 30 mV. Beyond this voltage, the conductivity of the pristine WZ system increases, to being approximately 13% larger than the pristine ZB system, from 0.2 to 0.7 V. At the end of this range, both ZB and WZ are beyond their electrical breakdown, due to the reduced band gap in these calculations. Because ZB has a lower band gap than WZ, the breakdown-induced current has a lower onset in ZB. After the onset of the breakdown current, the conductivity of systems increases rapidly. Since ZB has a lower onset than WZ, the conductivity of WZ falls drastically, in comparison, after the ZB onset. It can be seen, that the defect systems follow a similar trend. The defect systems that are most similar, to the pristine WZ system, have the most similar conductivity curve.

It is necessary, for InP as a channel material candidate, to investigate how the conductivity changes with the Fermi level. The Fermi level is usually controlled by the doping concentration, or the applied gate voltage. This is what is shown in Fig. 6b, with the Fermi level shift being represented, by the doping level necessary to achieve it. We see that increasing the n-type doping, is similar to increasing the bias-voltage beyond the Ohmic region, which means that, the defect systems conductivity increases similarly to the WZ system, depending on their similarity to the WZ system. On the p-doping side, the conductivity decreases slowly, until the doping reaches a level of approximately $4\times 10^{19}\; \hbox {cm}^{-3}$. After this level the conductivity falls sharply, to a mere 5% of the ZB value, for the pristine WZ system. The conductivity of the defect systems falls similarly, depending on their similarity to the WZ system. Despite having the lowest WZ fraction, the system with 1 RTP every 9 layers of ZB, does not have the highest conductivity in this region. This indicates that there is an upper limit in this doping region, whereafter further increasing the systems similarity to ZB, does not further increase the conductivity. This limit seems to have been reached in the systems with 6 layers of ZB, where the conductivity reaches a value of 38% of the pristine ZB system.

**Figure 7:**
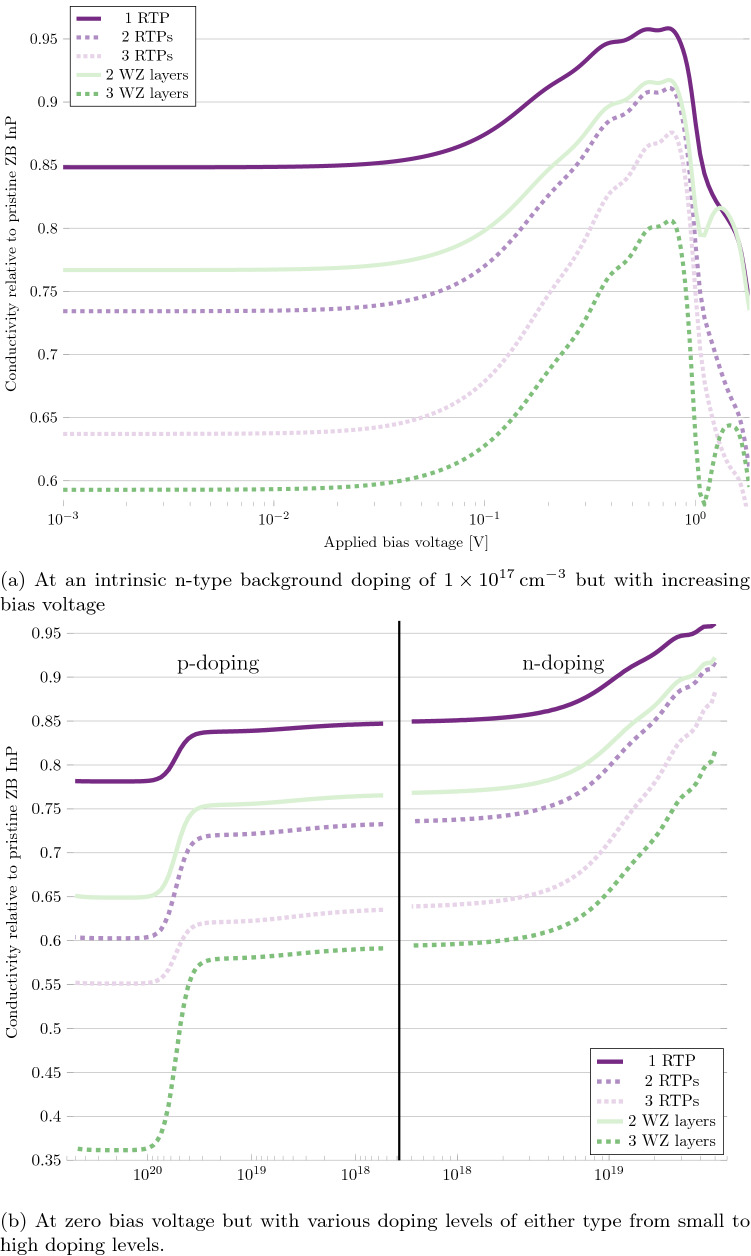
Differential conductivity of ZB InP with singular phase-defects along the [111]/[0001] crystallographic direction, relative to a pristine ZB system.

According to these results, the worst possible system is a system with a low frequency of defects. However, at low doping levels, a pristine ZB system has a higher conductivity than any of the defect systems. We would therefore expect, the conductivity to approach that of the pristine ZB system at some point, as the frequency of defects are lowered and the system is approaching the pristine system. To investigate this hypothesis, we set up systems of pristine ZB with singular defects. We set up three systems with 1, 2 and 3 RTPs in the central region, with a separation of 3 ZB layers between them. We also set up two systems with a single 2- or 3-layer WZ segment. The conductivity of these systems are shown in Fig. 7.

In Fig. 7a we see the same WZ conductivity behaviour as in the dense defect systems, with an increase in relative conductivity after the Ohmic region, followed by a sharp fall at approximately 0.9 V. Even in the extreme case of a single RTP, the closest possible defect system to pristine ZB, this behaviour is observed. The conductivity behaviour is likewise similar as a function of doping levels, as seen in Fig. 7b, with an increased conductivity with increasing n-type doping levels, and a sharp fall at a p-type doping level of $4\times 10^{19}\;\hbox {cm}^{-3}$. The WZ-like behaviour is less severe in these singular defect systems, as compared to the dense defect systems, which is seen by the reduced steepness of the conductivity curve at 0.9 V, and the smaller drop of the conductivity curve at a p-type doping level of $4\times 10^{19}\;\hbox {cm}^{-3}$. As could be expected, the WZ-like behaviour is strongest(weakest) in the most(least) WZ-like system, the system with a single WZ segment of 3 layers(a single RTP). Despite this WZ-like behaviour, which in the case of the dense defect systems actually increased the conductivity, all the investigated systems here exhibit a reduced conductivity and the reduction actually increases with the WZ-likeness. The least WZ-like system, the system with a single RTP, show a 15% reduction compared to pristine ZB, whereas the most WZ-like system, the system with a single 3-layer WZ segment, show a reduction of approximately 40%. Evidently, in the extrema of singular defects, the phase defects limits current transport along the [111]/[0001]-direction. Much more so than in the opposite extrema of highly intermixed ZB/WZ phases, where the current can actually be increased, relative to pristine ZB, at high n-type doping levels.

**Figure 8:**
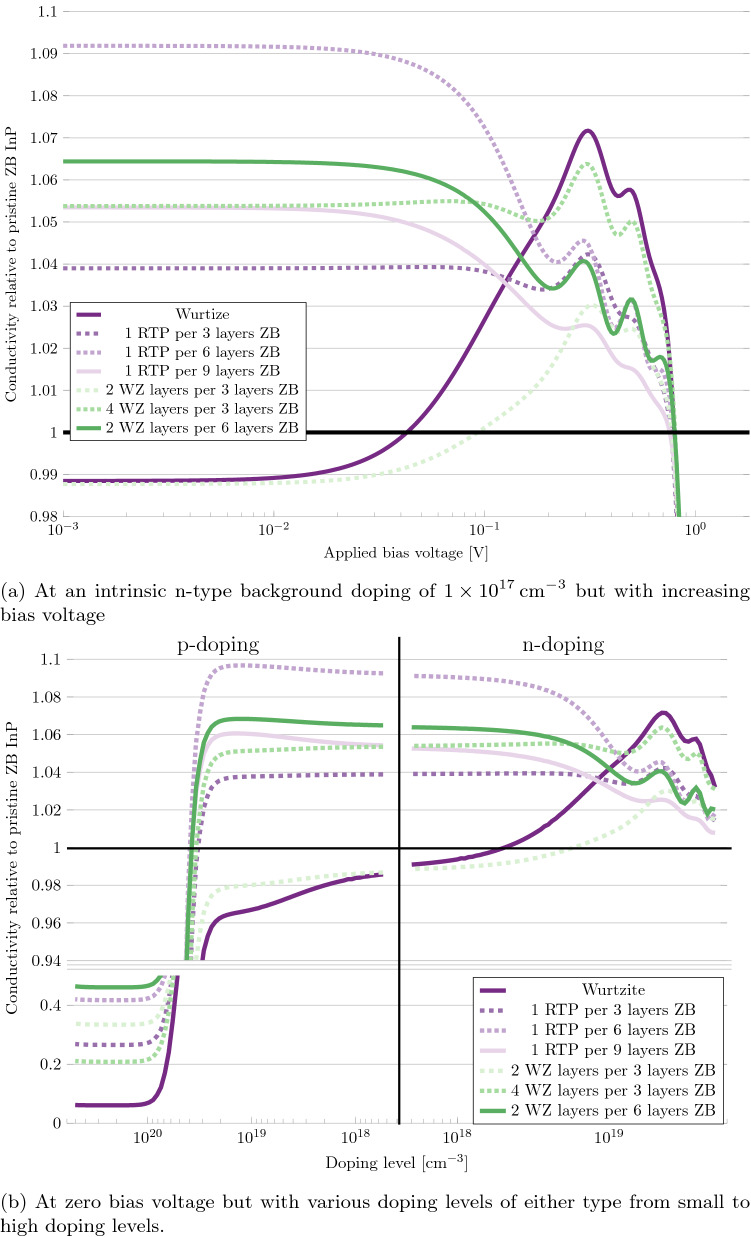
Differential conductivity of InP phase-defect systems along the [11$\bar{2}$]/[10$\bar{1}$0] crystallographic direction, relative to a pristine ZB system.

Considering the ZB/WZ anisotropy shown earlier in Fig. 3, and the clear tendency for the phase-defect systems to act WZ-like, it is of interest to investigate the conductivity anisotropy of the dense defect systems. Since the phase-defects are 2D defects, we expect a large anisotropy between conducting current across the defects ([111]/[0001]) or in-plane along the defects. We therefore repeated the conductivity calculations of the dense defect systems along the [11$\bar{2}$]/[10$\bar{1}$0]-direction, as this is a direction identical to the *M*-direction, which showed the highest transmission coefficient and smallest effective mass. These calculations are shown in Fig. 8.

In Fig. 8a we see that along the [11$\bar{2}$]/[10$\bar{1}$0]-direction, the conductivity of the pristine WZ system is almost identical, to the conductivity of the pristine ZB system in the Ohmic region. The conductivity of the WZ system increases by the same 8% as in the [111]/[0001]-direction, after the Ohmic region ends, reaching a value approximately 7% higher than the pristine ZB system just before 0.9 V. The most intermixed system of 2 layers of WZ per every 3 layers of ZB show a similar behaviour, with approximately half the increase of the WZ system. The remaining systems all show a vastly different behaviour than in the [111]/[0001]-direction, having higher conductivities along this crystallographic direction, in the Ohmic region, than both the pristine WZ and ZB system. The largest increase is seen in the system with 1 RTP every 6 layers of ZB, at a 9% increase in conductivity. We observe a general trend of larger increases in conductivity for smaller periodicities of defects. This trend falls off with the system with 1 RTP every 9 layers of ZB.

Based on the results in this section, it can be concluded that the defects function as pathways of lower resistance, thereby increasing the conductivity of the system. The reason why the increase in conductivity is higher in the systems with a lower fraction of defects, can be explained from the strain in the systems. As is well-known in the case of silicon, strain can increase conductivity. If this is the cause of the increased conductivity, there will be an optimum in the periodicity of the defects. Lowering the periodicity further below this optimum, will induce no additional stress, but reduces the number of pathways of reduced resistance for the electrons, thereby reducing the conductivity again. We find this optimum to be the system with 1 RTP every 6 layers of ZB, as the system with 1 RTP every 9 layers of ZB indeed has a lower conductivity. This also aligns with the strain on the RTP in the system. Reducing the periodicity from 1 RTP every 3 layers, to 1 RTP every 6 layers, induces an additional 0.07% strain (keep in mind that there is only a 0.37% strain between WZ and ZB in general in InP). Reducing the periodicity further, to 1 RTP every 9 layers of ZB, only increases the strain by an additional 0.003%, while reducing the number of RTPs by 33%. This defect-induced increase in conductivity starts to dissipate after the Ohmic region ends at 30 mV, whereafter the systems starts to act like in the [111]/[0001]-direction, showing a behaviour similar to WZ depending on the similarity of the system. Looking at Fig. 8b, we see that the defect-induced increase in conductivity, increases slightly during increasing p-doping, until the sudden drop at $4\times 10^{19}\;\hbox {cm}^{-3}$. At the n-type doping side it falls slightly with increasing doping, until it starts to dissipate completely after a doping level of $5\times 10^{18}\;\hbox {cm}^{-3}$. It should be noted that the increase in conductivity shown in Fig. 8, is relative to a pristine ZB system along [11$\bar{2}$]. The conductivity of the pristine ZB system is larger along [111], and none of the systems in Fig. 8 surpass this value, although the system with 1 RTP every 6 layers of ZB comes close to it.

## Discussion

In this work, we performed an ab initio investigation of InP polytypism, using a combination of DFT and NEGF methods, with the aim of discovering the impact of RTPs and phase-mixing on electrical properties, as well as the validity of these to work as quantum wells. Our simulations revealed that a minimal width of at least 10 nm is required, for a crystal-phase quantum well to reach the well-phase band gap value at the centre of the well. This result excludes RTPs as quantum wells, as well as the small polytypism usually encountered during uncontrolled growth processes. A crystal-phase quantum well in InP may still be possible through crystal-phase engineering, but they will not appear at random.

We also found that for conducting current along [111] in InP, it is desirable to have as many phase-defects as possible, so as to get as close to the WZ phase as possible. If the InP is p-doped, a slightly better conductivity can be achieved by pristine ZB InP, but having even a single phase-defect reduces conductivity substantially. Considering the likelihood of a single RTP forming, it is safer to aim for a high degree of phase-defects and accept the minimal reduction in conductivity. If the InP is n-type doped, having a high-degree of phase-defects even increase the conductivity. For conducting current along [11$\bar{2}$] in InP, we found that having a small fraction of phase-defects is desirable. We found that the phase defects act as pathways of lesser resistance for the current. These pathways becomes more conducting when under a higher strain from the otherwise pristine lattice. Therefore, an optimum exists, at which the phase defects appear in the highest possible number, while also being under the highest possible strain from the lattice. We found this optimum to be the system with a single RTP every 6 layers of ZB, since increasing the spacing between RTPs further, induces no substantial additional strain on the RTPs.

## Methods

In this study we used Density Functional Theory (DFT), which is an ab initio atomistic simulation method, in which the electron density is treated as the fundamental variable and is used to describe the system of interest. Atoms are treated explicitly through basis sets consisting of Linear Combinations of Atomic Orbitals (LCAO) and their corresponding pseudopotentials. DFT is in principle an exact method, if the exact form of the exchange-correlation functional was known. In reality, different levels of approximations are used which each have their own advantages and disadvantages. We also combined DFT with Non-Equiligrium Greens Functions (NEGF), when calculating conductivities or Local Density of States (LDOSs). In a NEGF calculation the system is split into 3 domains, the central region containing the system of interest, and two semi-infinite electrodes, which match with the left and right side of the central region respectively and work as electron reservoirs. The central region is then represented by Greens functions, which describes the propagation of electrons as well as their coupling to the electrodes. In contrast to regular DFT, with NEGF the system is only periodic in 2 dimensions, the last dimension, the transport direction, is handled by the semi-infinite electrodes, which are repeated infinitely in the left- and right-direction of the system respectively.

For the DFT and NEGF calculations in this study, we used the “High” version of the PseudoDojo^34^ basis sets and their corresponding pseudo-potentials, as implemented in the T-2022.03 version of QuantumATK. For the exchange and correlation functional, we mainly used a Generalized Gradient Approximation (GGA), made by Perdew, Burke and Ernzerhof especially to describe solids (PBES)^35^. We also used the more computationally expensive hybrid functional by Heyd, Scuseria and Ernzerhof (HSE06)^36^, to compare our results with and validate the use of the PBES functional. We sampled the Brillouin zone with a k-point density of 8Å for bulk calculations, which was increased to 150Å along the z-direction in the ZB/WZ interface calculations. In the NEGF calculations the sampling perpendicular to the transport direction, was increased to 27Å and along the transport direction it was 100Å. For transmission spectra calculations, the transverse samplings were increased to 48Å. The energy spacing used in the transmission spectra was 1.5 meV and in the PDOS calculations of the ZB/WZ interfaces it was 2.5 meV. We also used a real-space density mesh cutoff of 85 Ha and a Fermi-Dirac distribution at 300 K for the occupation. For the bulk calculation Boundary Conditions (BCs) we used periodic BCs, whereas we used Dirichlet BCs for the transport direction in the NEGF calculations.

All systems were constructed from minimal bulk InP systems, which were relaxed until the forces between the atoms were smaller than ${0.05}\;\hbox {eV }{\text{\AA} }^{-1}$ and the stress was less than 0.1 GPa. To construct the systems of interest, supercells of both phases were formed along the [111]/[0001] or [11$\bar{2}$]/[10$\bar{1}$0] directions, and put together to form RTPs or WZ/ZB interfaces. The systems were then relaxed again, to account for any defect-related stress, and converted to NEGF systems if necessary.

## Supplementary Information

Supplementary Information.

## Data Availability

The data from this study are available from the corresponding author upon request.

## References

[1] Bhol K, Nanda U, Jena B (2021). Journey of Mosfet from planar to gate all around: A review. Recent Pat. Nanotechnol..

[2] Hoyt, J. *et al.* Strained silicon MOSFET technology in Digest. In *International Electron Devices Meeting*, 23–26 (2002). 10.1109/IEDM.2002.1175770.

[3] Alamo J (2011). Nanometre-scale electronics with III–V compound semiconductors. Nature.

[4] Del Alamo, J. A. *et al.* III–V MOSFETs for future CMOS. In *2015 IEEE Compound Semiconductor Integrated Circuit Symposium (CSICS)*, 1–4 (2015). 10.1109/CSICS.2015.7314512.

[5] Heyns M, Tsai W (2009). Ultimate scaling of CMOS logic devices with Ge and III–V materials. MRS Bull..

[6] Smit M (2014). An introduction to InP-based generic integration technology. Semicond. Sci. Technol..

[7] Jiao Y (2020). InP membrane integrated photonics research. Semicond. Sci. Technol..

[8] Mokkapati S, Jagadish C (2009). III–V compound SC for optoelectronic devices. Mater. Today.

[9] Stillman G, Robbins V, Tabatabaie N (1984). III–V compound semiconductor devices: Optical detectors. IEEE Trans. Electron Devices.

[10] Zhao H (2018). Topological hybrid silicon microlasers. Nat. Commun..

[11] Staudinger P (2020). Wurtzite InP microdisks: From epitaxy to roomtemperature lasing. Nanotechnology.

[12] Bioud Y (2019). Uprooting defects to enable high-performance III–V optoelectronic devices on silicon. Nat. Commun..

[13] Krishnamachari U (2004). Defect-free InP nanowires grown in [001] direction on InP (001). Appl. Phys. Lett..

[14] Joyce HJ (2007). Twin-free uniform epitaxial GaAs nanowires grown by a two-temperature process. Nano Lett..

[15] Tahini H, Chroneos A, Murphy S, Schwingenschlögl U, Grimes R (2013). Vacancies and defect levels in III–V semiconductors. J. Appl. Phys..

[16] Moram MA, Oliver RA, Kappers MJ, Humphreys CJ (2009). The spatial distribution of threading dislocations in gallium nitride films. Adv. Mater..

[17] Chen Y (2016). Effect of a high density of stacking faults on the Young’s modulus of GaAs nanowires. Nano Lett..

[18] Bao J (2008). Optical properties of rotationally twinned InP nanowire heterostructures. Nano Lett..

[19] Mahajan S (2000). Defects in semiconductors and their effects on devices. Acta Mater..

[20] Stamp C, Van de Walle CG (1998). Energetics and electronic structure of stacking faults in AlN, GaN, and InN. Phys. Rev. B.

[21] Rebane Y, Shreter Y, Albrecht M (1997). Stacking faults as quantum wells for excitons in wurtzite GaN. Phys. Status Solidi a.

[22] Corfdir P (2014). Stacking faults as quantum wells in nanowires: Density of states, oscillator strength, and radiative efficiency. Phys. Rev. B.

[23] Korona K (2014). Dynamics of stacking faults luminescence in GaN/Si nanowires. J. Lumin..

[24] Albrecht M (1997). Luminescence related to stacking faults in heterepitaxially grown wurtzite GaN. MRS Online Proc. Lib. Arch..

[25] Smidstrup S (2020). QuantumATK: An integrated platform of electronic and atomic-scale modelling tools. J. Phys. Condens. Matter.

[26] Smidstrup S (2017). First-principles Green’s-function method for surface calculations: A pseudopotential localized basis set approach. Phys. Rev. B.

[27] Brandbyge M, Mozos J-L, Ordejóon P, Taylor J, Stokbro K (2002). Densityfunctional method for nonequilibrium electron transport. Phys. Rev. B.

[28] Staudinger P, Mauthe S, Moselund KE, Schmid H (2018). Concurrent zinc-blende and Wurtzite film formation by selection of confined growth planes. Nano Lett..

[29] De A, Pryor CE (2010). Predicted band structures of III–V semiconductors in the wurtzite phase. Phys. Rev. B.

[30] Vurgaftman I, Meyer JR, Ram-Mohan LR (2001). Band parameters for III–V compound semiconductors and their alloys. J. Appl. Phys..

[31] Signorello G (2014). Inducing a direct-to-pseudodirect bandgap transition in wurtzite GaAs nanowires with uniaxial stress. Nat. Commun..

[32] Pavesi L, Piazza F, Rudra A, Carlin JF, Ilegems M (1991). Temperature dependence of the InP band gap from a photoluminescence study. Phys. Rev. B.

[33] Dacal L, Cantarero A (2016). An ab initio study of the polytypism in InP. Sci. Rep..

[34] Van Setten M (2018). The PseudoDojo: Training and grading a 85 element optimized norm-conserving pseudopotential table. Comput. Phys. Commun..

[35] Perdew JP, Burke K, Ernzerhof M (1996). Generalized gradient approximation made simple. Phys. Rev. Lett..

[36] Heyd J, Scuseria GE, Ernzerhof M (2003). Hybrid functionals based on a screened Coulomb potential. J. Chem. Phys..

