# Supplementary material for: First-principles investigation of polytypic defects in InP
Source: Sci Rep. 2022 Nov 16;12:19724. doi: 10.1038/s41598-022-24239-w (PMC9669039; doi:10.1038/s41598-022-24239-w)
Supplement: Supplementary file 1 — Supplementary Information. [file 41598_2022_24239_MOESM1_ESM.pdf]

9 Supplementary information

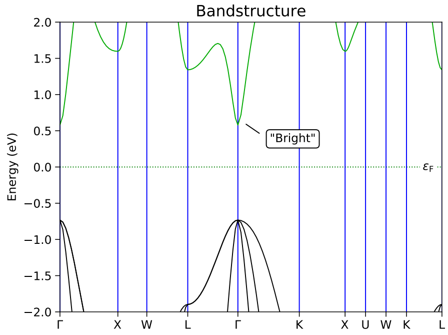

(a) ZB minimal unit cell

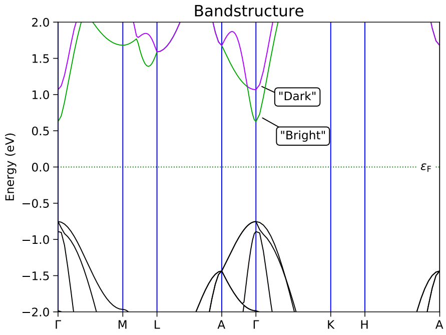

(b) WZ minimal unit cell

Figure 9 Band structures of InP phases in their minimal unit cells calculated with HSE06

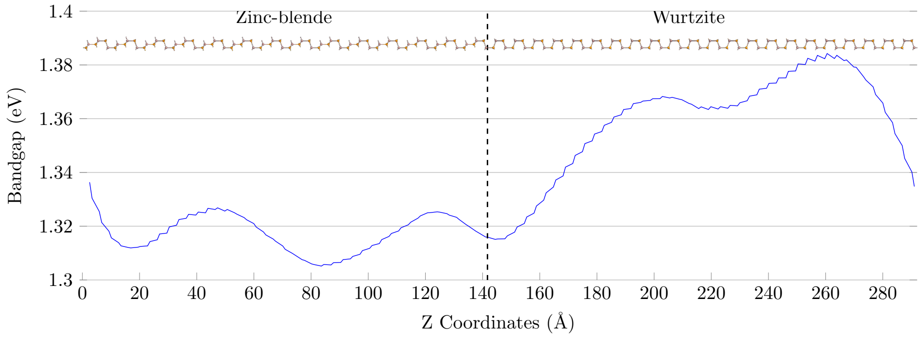

Figure 10 Conduction-Valence band energy difference in 86 atomic layer long InP system calculated with HSE06

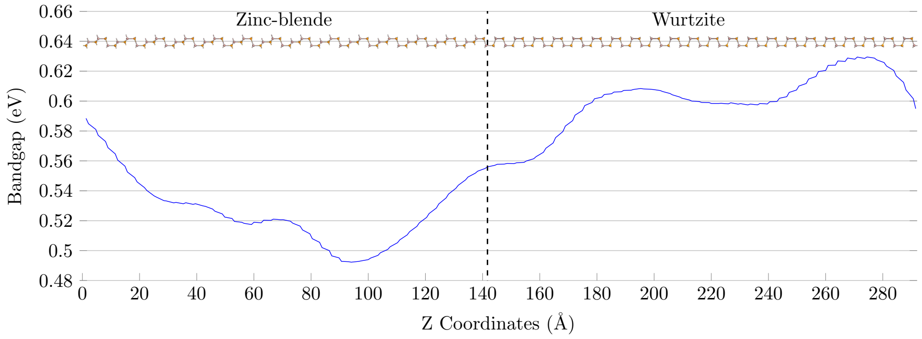

Figure 11 Conduction-Valence band energy difference in 86 atomic layer long InP system calculated with GGA
